# Association between regional brain volumes and BMI z-score change over one year in children

**DOI:** 10.1371/journal.pone.0221995

**Published:** 2019-09-19

**Authors:** Travis D. Masterson, Carly Bobak, Kristina M. Rapuano, Grace E. Shearrer, Diane Gilbert-Diamond

**Affiliations:** 1 Department of Epidemiology, Norris Cotton Cancer Center, Geisel School of Medicine at Dartmouth College, Hanover, New Hampshire, United States of America; 2 Department of Biomedical Data Science, Geisel School of Medicine at Dartmouth College, Hanover, New Hampshire, United States of America; 3 Department of Psychology, Yale University, New Haven, Connecticut, United States of America; 4 Department of Nutrition, University of North Carolina at Chapel Hill, Chapel Hill, North Carolina, United States of America; Universidade de Sao Paulo, BRAZIL

## Abstract

**Purpose:**

Associations between brain region volume and weight status have been observed in children cross-sectionally. However, it is unclear if differences in brain region volume precede weight gain.

**Methods:**

Two high-quality structural brain images were obtained approximately one year apart in 53 children aged 9–12 years old. Children’s height and weight were also measured at each scan. Structural images were processed using the FreeSurfer software-package providing volume measures for regions of interest including the entorhinal cortex, nucleus accumbens, and hippocampus. Age- and sex-adjusted BMI z-scores (BMIz) were calculated at both timepoints. The association between brain region volume and BMIz was examined cross-sectionally using linear regression and longitudinally using structural equation modeling. All models were adjusted by estimated cranial volume to account for individual variation in head size and were corrected for multiple comparisons (pFDR<0.05).

**Results:**

The sample of children was primarily healthy weight at baseline (79.78%). Cross-sectionally at the one-year follow-up, a positive relationship was observed between right hippocampal volume and BMIz (*β* = 0.43, 95% *CI* = (0.10, 0.77)). Longitudinally a negative relationship was observed between right entorhinal volume at baseline and BMIz at the one-year follow-up (*β* = −0.25, 95% CI = (−0.44, −0.07)).

**Conclusion:**

These results suggest that measured volumes from certain regions of the brain that have been associated with BMI in adults are associated with both concurrent BMIz and BMIz change over one-year in a primarily healthy weight sample of children. As the entorhinal cortex integrates signals from both reward and control regions, this region may be particularly important to weight management during child development.

## Introduction

Rates of childhood obesity continue to rise in the United States [1]. Obesity is related to a host of co-morbidities that affect long term health outcomes as well as overall quality of life [2]. Increased weight has also been shown to be associated with altered brain morphology (i.e., gray matter density and/or regional volume) [3–6]. Associations between the morphometry of developing brain regions and adiposity during childhood and adolescence are of interest because significant growth and remodeling of gray matter occurs during this period [7], and because the emergence of overweight in youth often tracks into adulthood [8]. Furthermore, a recent study has highlighted that brain region volume is particularly related to body weight and body mass index z-scores (BMIz) in children [3]. Therefore, volumetric analysis may be particularly useful for understanding which regions of the brain are developmental important to weight gain and weight management over childhood and adolescence.

The nucleus accumbens has been previously implicated in responding to environmental food cues [9–11]. Increased response to food cues in this region has been shown to correlate with increased weight gain in young adults (18-19-year old participants) [12]. Several studies have examined nucleus accumbens volume in relation to body mass index (BMI) and have found that larger nucleus accumbens volumes tend to correlate with higher BMI in adults [13,14] and BMIz in children [3]. A recent study by Rapuano et al. has also shown that nucleus accumbens volume is associated with genetic risk for the development of obesity in young children [11]. Given these observed associations, we sought to explore the association between nucleus accumbens volume and future adiposity gain in children. Further, animal studies [9] have shown that the nucleus accumbens works in concert with other regions that have been cross sectionally correlated with BMI in both adults and children such as the entorhinal cortex [15] and hippocampus [16]. As both the entorhinal cortex and hippocampus are thought to be particularly important for conditioned learning, particularly in relation to latent inhibition, these regions may also be predictive of future weight gain [17]. For example, Burger and Stice (2014) have demonstrated that food-cue related reward learning is predictive of future weight gain in adults [18].

For example, the entorhinal cortex is thought to act as a gatekeeper between brain regions including the nucleus accumbens and the hippocampal formation [19]. A recent analysis was conducted in a large brain imaging cohort of adults (n = 895; mean ± SD = 28 ± 3.67 years) using volumetric brain assessment to examine the relationship between the volume of brain structures located in the medial temporal lobe and BMI [15]. This study found that, bilaterally, the entorhinal cortex was negatively associated with BMI [15]. However, it is unclear if the relationship between entorhinal volume and BMI observed in adults is also apparent in younger populations. Therefore, this region holds interest for examination of both cross-sectional as well as longitudinal relationships with adiposity.

The hippocampus is also of particular interest. Not only is it important for conditioned learning it is also consistently observed to functionally respond to environmental food cues and is thought to play a key role in the regulation of energy intake [10,11,16,20–22]. Structurally, the hippocampus receives input from regions of the brain associated with reward such as the nucleus accumbens and brain regions responsible for appetite regulation such as the hypothalamus [23,24]. However, volumetric analyses of the hippocampus in child and adolescent populations have provided mixed results, with some studies showing a positive relationship between hippocampal volume and BMIz [6], some showing a negative relationship[16,25,26], and others showing no relationship [3]. Therefore, similar to the nucleus accumbens and entorhinal cortex, this region holds interest for both cross-sectional and longitudinal analyses in relation to adiposity.

Although previous assessments of brain morphology in relation to BMI provide insights into potential neural associations with adiposity, the lack of longitudinal data limits their interpretation. Longitudinal studies are particularly important for understanding volumetric associations in younger populations who are experiencing rapid changes in height, weight, and brain development. As BMIz has been shown to be the most optimal measure of annual adiposity change in elementary school children we focused on this metric as our main outcome measure [27]. Therefore, the purpose of this study was to assess the relationship between volumetric measures of the entorhinal cortex, hippocampus, and nucleus accumbens and child adiposity using repeated measurements at two time points approximately one year apart. Based on previous studies, we hypothesized that entorhinal cortex [15] and hippocampal [16] volume would negatively correlate with concurrent BMIz at both baseline and follow up, whereas nucleus accumbens volumes would positivity correlate [3]. Similarly, we hypothesized that entorhinal cortex and hippocampal volumes would be negatively associated with a change in BMIz between time point 1 and time point 2, while nucleus accumbens volume would show a positive relationship.

## Methods

### Participants

Seventy-eight (*N* = 78; 42 male) children between the ages of 9 and 12 years (mean ± SD = 10.3 ± 0.8 years) were recruited as a sub-sample of a larger study (n = 200) through fliers placed throughout the Upper Valley community and a contact list from the Children’s Hospital at Dartmouth. Participants recruited in this sub-sample were all right handed, native English speakers, and reported normal neurological history. Dartmouth’s Committee for Protection of Human Subjects institutional review board approved all study protocols. Each child provided written assent and parents provided informed consent. All participants received monetary compensation for participating. As part of this follow up study the 78 participants completed a neuroimaging study, which included a structural magnetic resonance imaging (sMRI) scan [11]. Of the 78 participants, ten sMRI scans were removed due to low signal-to-noise ratios and two were removed due to inadequate parcellation of anatomical structures determined by visual and statistical inspection, resulting in 66 high-quality scans at the first timepoint. Of the original 78 participants, 65 returned approximately 1-year later for a follow-up visit. Six participants were unable to complete the follow-up scan due to newly acquired braces or metal retainers, resulting in a total of 59 follow-up sMRI scans obtained at the second timepoint. Of these 59 sMRI scans, six were removed due to low signal-to-noise ratios and inadequate parcellation of anatomical structures. Thus, a total of 53 individuals had usable data at time 2. Overall, 47 participants had viable scans at both time 1 and time 2 of this analysis.

### BMI and BMI z-score

Participants heights and weights were collected prior to their MRI scans at both visits. Study staff assessed heights using a calibrated professional-grade stadiometer (model Seca 216) to a tenth of a centimeter. Participant weight was measured on a calibrated Tanita scale (model TBF-300A). Both measurements were collected once using standardized procedures. Child BMI was calculated based on participants’ height (m^2^) and weight (kg). Age and sex adjusted BMI z-scores were calculated based on the Center for Disease Control 2000 growth standards.[28]

### Pubertal assessment

Puberty was self-reported by participants using the Self-Rating Scale for Pubertal Development.[29] This questionnaire consists of three standard questions (growth spurt, body hair, skin changes), two specific questions for boys (voice deepening, facial hair) and two specific questions for girls (breast growth, menstruation). Each question is answered on a scale of 1–4 anchored at “has not yet begun” to “seems completed” apart from menstruation which is scored either as a 1 (no) or 4 (yes). Three questions (body hair and the two sex specific questions) are used to categorize children into one of five pubertal statuses including pre, early, mid, late, or completed puberty [30]. Seven of the 78 children did not complete the pubertal assessment therefore the average pubertal status of the appropriate sex-specific strata were used.

### Structural MRI image acquisition

Structural scanning was performed on a 3T Philips Achieve MRI fit with a 32-channel SENSE (Sensitivity Encoding) head coil. Structural images were obtained using a T1-weighted magnetization prepared rapid gradient echo (MPRAGE) protocol [repetition time (TR) = 9.9 ms; echo time (TE) = 4.6 ms; flip angle = 8°; 1 × 1 × 1-mm^3^ voxels] at both time points.

### Structural image preprocessing and volume assessment

Structural images were reconstructed and segmented using FreeSurfer (5.3.0), an automated segmentation tool that has been demonstrated to label structures comparably to manual tracing techniques [31]. FreeSurfer’s quality assurance tool, QAtools, allows for quantitative assessment of data quality [32,33] and was used to calculate signal-to-noise ratios and statistical outliers for each segmentation completed. A series of screenshots were also generated by the QA tool from various steps throughout the reconstruction pipeline to allow rapid visual inspection of segmentation quality for each participant. As variation in image quality has been shown to be particularly important to morphometric assessment [34] structural images that were one standard deviation below the sample mean (17.1±2.6 at time point 1 and 17.0±2.0 at time point 2) or that were extreme statistical outliers (morphometric values >3 times the interquartile range) or those that did not pass standard visual inspection were excluded from the analysis. This extensive quality control procedure was used to ensure only high-quality segmentations were obtained as movement during scanning is quite common which leads to reduced quality of underlying structural images [35]. To account for variation in overall cranial size, estimated intracranial volumes were extracted for use in all statistical models. Finally, to increase interpretability, estimated brain volumes and intracranial volumes were z-standardized within the sample. Statistical models were run using both standardized and unstandardized values to ensure transformations did not alter the interpretation of results.

### Statistical analysis

All statistical analyses were conducted using R version 3.4.3. To initially assess the concurrent relationship between BMI z-score and region volumes at either baseline or follow up, linear regression models were calculated with BMI z-score as the dependent variable and region volume at the associated time point as an independent variable. Individual models were used for each brain region. Intracranial volume, age, sex, and pubertal status were entered in to the regression as independent variables to control for these variables in the model. Sex-specific analyses were also conducted (see S1 and S2 Tables).

**Fig 1:**
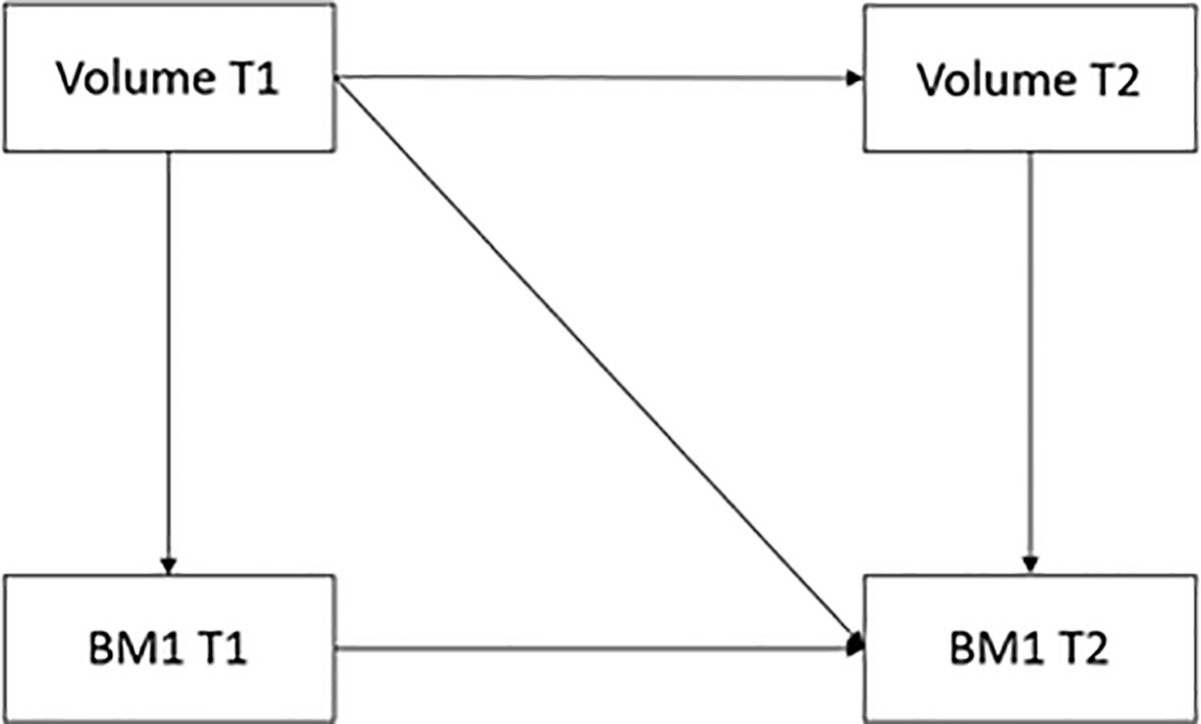
Graphical representation of the structural equation model used to assess the longitudinal relationship between brain region volume and BMI z-score.

To further elucidate the temporal relationship between BMIz and *a priori* defined brain region volumes, a structural equation model (SEM) was implemented using the ‘*lavaan’* package.[36,37] SEM modeling allows for the estimation of the association between brain region volume and BMIz while controlling for the stability of the variables over time and accounting for covariation between the variable parameters [38,39]. As we were primarily interested in examining the hypothesis that brain region volume is predictive of BMIz based on previous research [3,11], our SEM paths are designed to test if current and previous brain region volumes are predictive of BMIz (Fig 1).

Only those samples with complete data (both time 1 and time 2) were used, for a total of n = 47 in the SEM model. A generalized least squares estimator was used to fit each of the models. As in the linear regression models, individual models were computed for each brain region. Models were adjusted for age at current timepoint, sex, puberty score, and intracranial volume (Eq 1).

$$\begin{array}{c} Region_{t_{2}}=\beta_{1}\times Region_{t_{1}}+\beta_{2}\times CranialVolume_{t_{1}}+\beta_{3}\times Age_{t_{2}}+\beta_{4}\times Sex+\beta_{5}\times Puberty+\zeta_{Region1}\\ BMI_{t_{1}}=\beta_{6}\times Region_{t_{1}}+\beta_{7}\times CranialVolume_{t_{1}}+\beta_{8}\times Age_{t_{1}}+\beta_{9}\times Sex+\beta_{10}\times Puberty+\zeta_{{BMI}_{t_{1}}}\\ BMI_{t_{2}}=\beta_{11}\times BMI_{t_{1}}+\beta_{12}\times {Region}_{t_{1}}+\beta_{13}\times {CranialVolume}_{t_{1}}+\beta_{14}\times {Region}_{t_{2}}+\beta_{15}\times {CranialVolume}_{t_{2}}+\beta_{16}\times Age_{t_{2}}+\beta_{17}\times Sex+\beta_{18}\times Puberty+\zeta_{{BMI}_{t_{2}}} \end{array}$$ Eq 1

Due to the exploratory nature of this analysis as well as small sample size, covariates in the structural equation models were iteratively assessed while removing the variable with the largest p-value until all variables included had a p-value of 0.1 or less. This was done to improve overall model fit, as measured using a chi-square goodness of fit test, by removing variables that had little impact on the overall model. To maintain interpretability, individual brain regions of interest and total intracranial volume remained in all models. Model significance was assessed using an alpha of 0.05. To account for multiple comparisons, *p*-values in the SEM analysis were adjusted using Benjamini-Hochberg correction (q = 0.1; total comparisons = 12 [6 regions x 2 timepoints]) for an overall pFDR<0.05.

## Results

Sex stratified participant characteristics and brain volumes are summarized in Table 1. In the total sample the majority of families had at least one parent that had completed college or graduate school (89.8%) and the majority of families had above average household income (75.6% of households >$65,000/year). The sample at time point 1 (n = 66) included 39 males (59.0%) and 27 females (41.0%) and at time point 2 (n = 53) included 29 males (54.7%) and 24 females (45.3%). The longitudinal sample (n = 47) including 27 males (57.4%) and 20 females (42.6%). The mean child age at time point one was 10.40 years (SD = 0.76; range:9.33–12.92) and 11.86 years (SD = 0.81; range:10.25–13.53) at time point 2. Mean BMIz was 0.34 (SD = 0.95; range = -2.34–2.37) at time point 1 and 0.34 (SD = 1.03; range = -2.15–2.30) at time point 2. In the total sample 52 of the 66 children (78.78%) were considered healthy weight. Only one relationship was apparent in the cross-sectional analysis, a correlation between right hippocampal volume at time 2 and child BMIz at time 2 (*β* = 0.43, 95% *CI* = (0.10, 0.77)). None of the other brain regions tested showed significant cross-sectional associations. The unstandardized model estimates for the remaining cross-sectional analyses are summarized in Table 2.

**Table 1:** Participant characteristics stratified by sex.

	Males (n = 39)	Females (n = 27)		
	N (%)	N (%)	Chi-Square (*X*^*2*^*)*	p-value
**Age (Time 1)**				
9	6 (16.7)	3 (11.1)		
10	18 (50.0)	9 (33.3)		
11	12 (33.3)	12 (44.5)		
12	0 (0.0)	3 (11.1)	2.74	0.43
**Puberty (Time 2)**				
1 = Not Started	7 (17.9)	1 (3.7)		
2 = Early	28 (43.6)	17 (63.0)		
3 = Mid	4 (10.3)	2 (7.4)		
4 = Late	0 (0.0)	7 (25.9)		
5 = Completed	0 (0.0)	0 (0.0)	**13.10**	**0.004**
**Weight Status (Time 1)**				
Healthy Weight	27 (69.2)	25 (92.5)		
Overweight/Obese	12 (30.7)	2 (7.4)	**10.15**	**0.001**
**Brain Volumes**	Mean ± SD	Mean ± SD	t-value	p-value
L. Nucleus Accumbens Time 1	91.41 ± 13.18	87.70 ± 11.51	1.18	0.24
R. Nucleus Accumbens Time 1	87.05 ± 10.43	81.39 ± 8.47	**2.33**	**0.02**
L. Entorhinal Cortex Time 1	180.26 ± 32.59	167.52 ± 40.60	1.41	0.16
R. Entorhinal Cortex Time 1	164.07 ± 32.20	142.98 ± 29.43	**2.70**	**0.009**
L. Hippocampus Time 1	415.22 ± 38.05	401.21 ± 40.46	1.43	0.15
R. Hippocampus Time 1	419.92 ± 31.38	395.56 ± 37.19	**2.87**	**0.006**
Estimated Intracranial Time 1	144482.34 ± 13090.61	134323.52 ± 11074.01	**3.29**	**0.001**
L. Nucleus Accumbens Time 2	86.42 ± 13.05	88.26 ± 16.45	0.45	0.65
R. Nucleus Accumbens Time 2	84.13 ± 9.03	82.69 ± 11.48	0.50	0.61
L. Entorhinal Cortex Time 2	200.62 ± 36.27	179.38 ± 33.73	**2.19**	**0.03**
R. Entorhinal Cortex Time 2	187.50 ± 39.18	173.95 ± 28.41	1.36	0.17
L. Hippocampus Time 2	413.44 ± 35.94	407.92 ± 48.88	0.47	0.63
R. Hippocampus Time 2	422.05 ± 31.66	411.74 ± 43.45	0.99	0.32
Estimated Intracranial Time 2	143550.51 ± 14407.51	133819.89 ± 13918.93	**2.48**	**0.01**

All volume values expressed have been converted from mm^3^ to cm^3^

Chi-Square and t-tests conducted to test for differences between males and females

OW/OB = Children with overweight or obesity; Healthy weight = ≤85^th^ and OW/OB = >85 BMI-age-sex-percentile

Sample size at time 2: n = 53 (29 male)

Time 1 = Baseline; Time 2 = 1 Year Follow Up

**Table 2:** Adjusted linear regressions of BMIz on concurrent regional brain volumes at times 1 and 2.

Regression	Brain Region	Model Estimate β (95% CI)	
Baseline		Left	Right
	Nucleus Accumbens	0.11 (-0.13, 0.35)	0.06 (-0.20, 0.32)
	Entorhinal Cortex	-0.09 (-0.34, 0.14)	-0.21 (-0.47, 0.04)
	Hippocampus	-0.07 (-0.41, 0.25)	0.23 (-0.10, 0.56)
1-year Follow Up			
	Nucleus Accumbens	0.13 (-0.17, 0.43)	0.10 (-0.22, 0.43)
	Entorhinal Cortex	-0.27 (-0.59, 0.04)	-0.14 (-0.43, 0.14)
	Hippocampus	0.16 (-0.18, 0.51)	**0.43 (0.10, 0.77)** *

* Indicates p<0.05.

Models include control variables for estimated intracranial volume, age, sex, and pubertal status.

Volumes are reported as sample standardized z-scores = Volume–Sample Mean Volume / Sample Standard Deviation.

Sample size at time 1: n = 66 (39 male); Sample size at time 2: n = 53 (29 male).

**Fig 2:**
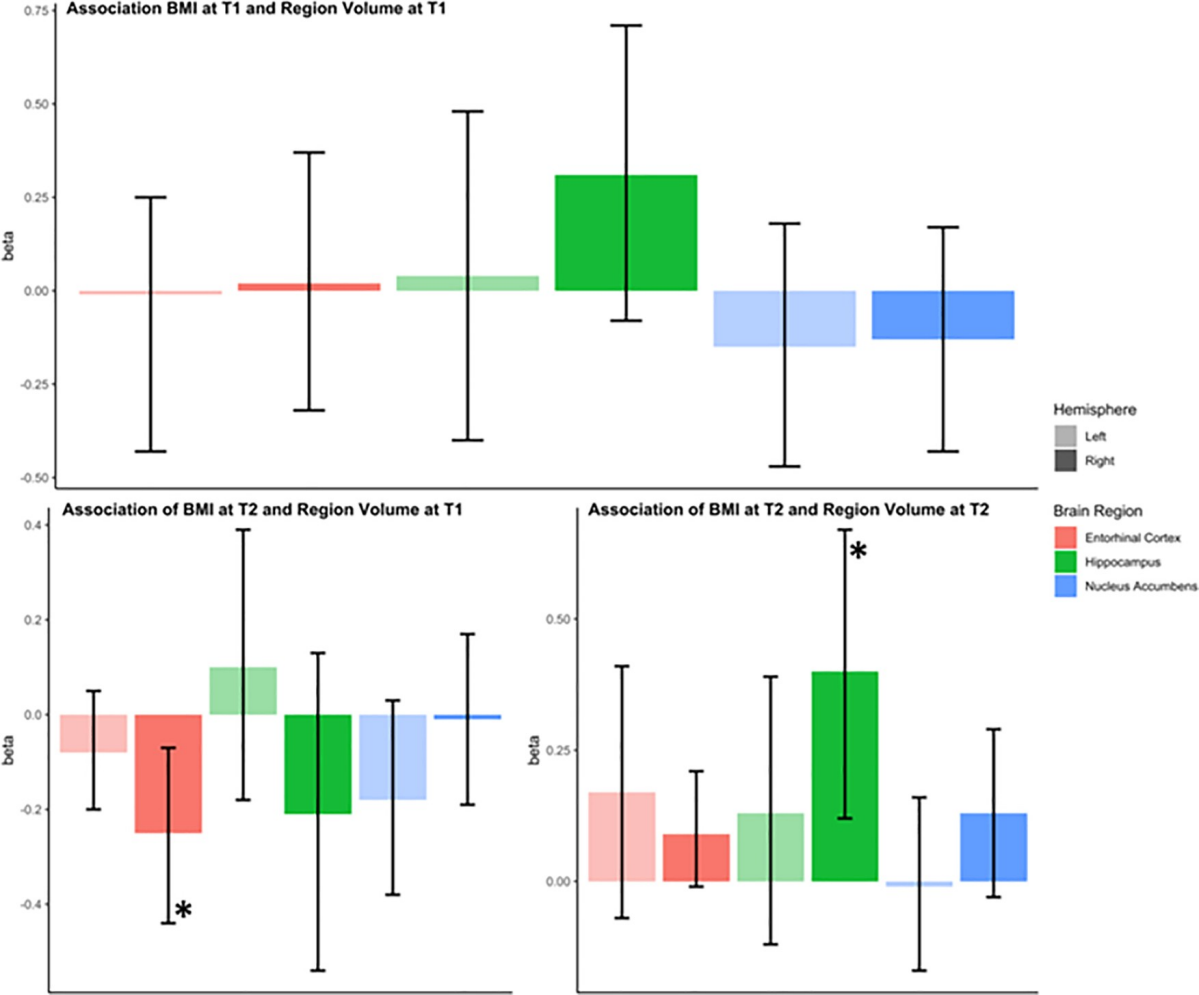
Graphical representation of the beta values derived from the structural equation model (SEM) for each brain region of interest. Error bars indicate 95% confidence interval. T1 = Baseline; T2 = 1 Year Follow Up. * indicates *p*<0.05.

The longitudinal SEM analysis identified a negative association between the right entorhinal cortex volumes at time point 1 and BMIz at time point 2 (one-year follow-up) (*β* = −0.25, 95% CI = (−0.44, −0.07)). A positive association was identified between the right hippocampal volume at time point 2 and concurrent BMIz at time point 2 (*β* = 0.40, 95% *CI* = (0.12, 0.67)) replicating the result seen in the cross-sectional model. No other relationships were apparent from the longitudinal analysis. The main effects from the SEMs between BMIz scores and regional brain volumes at baseline and one-year follow up are summarized in Table 3 and visualized in Fig 2.

**Table 3:** Regressions from the SEMs between BMIz scores and regional brain volumes at baseline and 1-year follow up.

Outcome	Brain Region Predictor	Model Estimate β (95% CI)	
BMIz at Time 1		Left	Right
	Nucleus Accumbens T1	-0.15 (-0.47,0.18)	-0.13 (-0.43,0.17)
	Entorhinal Cortex T1	-0.01 (-0.43,0.25)	0.02 (-0.32, 0.37)
	Hippocampus T1	0.04 (-0.40,0.48)	0.31 (-0.08,0.71)
BMIz at Time 2			
	Nucleus Accumbens T1	-0.18 (-0.38,0.03)	-0.01 (-0.19,0.17)
	Entorhinal Cortex T1	-0.08 (-0.20,0.05)	-0.25 (-0.44, -0.07)*
	Hippocampus T1	0.10 (-0.18,0.39)	-0.21 (-0.54,0.13)
	Nucleus Accumbens T2	-0.01 (-0.17,0.16)	0.13 (-0.03,0.29)
	Entorhinal Cortex T2	0.17 (-0.07,0.41)	0.09 (-0.01,0.21)
	Hippocampus T2	0.13 (-0.12,0.39)	0.40 (0.12,0.67)*

*Indicates Benjamini–Hochberg corrected p<0.05.

N = 47 (27 male)

Models include control variables for estimated intracranial volume, age, sex, and pubertal status where significant

Volumes are reported as sample standardized z-scores = Volume–Sample Mean Volume / Sample Standard Deviation.

T1 = Baseline; T2 = 1 Year Follow Up.

## Discussion

The purpose of the current analysis was to determine the cross-sectional and longitudinal relationships between child BMIz and volumetric measures of the entorhinal cortex, hippocampus, and nucleus accumbens. We had hypothesized that nucleus accumbens volume would be positively associated with BMIz both cross-sectionally and prospectively. Our hypothesis was motivated by a recent study in children that observed that nucleus accumbens volume was positively correlated with concurrent BMIz, [3] as well as our previous finding in our larger study that nucleus accumbens volume was positively associated with genetic risk for developing obesity as measured by *FTO* rs9939609 [11]. However, in the current analysis, we observed no cross-sectional or longitudinal relationship between nucleus accumbens and BMIz. Our small sample size in the current study, particularly within the SEM, prohibited the examination of genetic risk factors in relation to brain volume.

We did not find evidence to support the hypotheses that entorhinal cortex volume was cross-sectionally associated with BMIz in children, however, we did find that entorhinal cortex volume was negatively associated with changes in BMIz longitudinally. Therefore, while our findings do not support cross-sectional results like those found in adults[4] it does suggest a negative longitudinal relationship between entorhinal cortex volume and increases in BMIz over a one-year period within this small sample. The lack of replication of the cross-sectional relationship between entorhinal volume and BMIz may be in part due to the limited number of children with overweight or obesity in our sample or our sample’s younger age range. As the prevalence of obesity in early childhood is lower than that of adulthood [40], it is possible that a small difference in weight gain related to volume may not be easily observable as a cross-sectional association until adulthood, when excess weight has more time to accrue. Therefore, our results, alongside those of Vainik and colleagues, suggest that future studies should give more consideration to the entorhinal cortex as a region of interest particularly if study outcomes are related to BMIz.

Based on previous studies, we hypothesized that hippocampal volumes would negatively correlate with BMIz [6,41,42]. We further hypothesized hippocampal volume would be negatively associated with changes in BMIz. Using SEM, we did not observe any prospective relationship within our sample. However, contrary to our initial hypothesis we did observe a positive association between concurrent right hippocampal volume and BMIz at timepoint 2 in both cross-sectional and SEM results. Our cross-sectional findings add support to those previously reported in adolescents [6] however, we note that there are conflicting results in the current literature with some studies showing no relationship [3,4] and others observing the opposite relationship to that observed in this study [16,25,43]. The lack of a relationship with BMIz at baseline may suggest that BMIz-related associations with this region do not emerge until later in development. This relationship should be clarified by future studies employing large-scale longitudinal designs.

While these results provide novel insight into potential relationships between pediatric obesity and structural brain development, the current analyses include several limitations that should be considered. Although the sample size reported here is comparable to other recent analyses [3], the number of participants may be underpowered to detect significant relationships, or alternatively, may overestimate the significance of positive tests. More specifically our sample was underpowered for full SEM analysis. Therefore, these results should be viewed as exploratory and further follow up is needed to confirm our findings. Further, our sample was largely homogeneous in terms of BMI z-scores and other demographic characteristics such as education and income, which may reduce generalizability and may have limited our ability to detect cross-sectional relationships with BMIz. Primarily, the fact that the sample was primarily healthy weight likely limits the robustness of the overall results. Additionally, height and weight measurements were only assessed once during each visit, as opposed to duplicate/triplicate assessments and this may have introduced some small amount of measurement error. However, this analysis has several strengths. The first is that both brain volumetric data and BMIz scores were measured at two time points separated by a year. Second, our structural equation models allowed for analysis of two timepoints while simultaneously controlling for covariance between the two measures of BMIz and brain volumes. This type of modeling also has advantages over other types of modeling that could be employed, such as linear mixed models, as it provides statistical indications of causality. Finally, by employing both visual inspection and quantitative assessment of FreeSurfer segmentation, we assured only high-quality segmentation data were included in the analysis.

In summary, we observed that right entorhinal cortex volume negatively correlated with BMIz change over one year in children. Furthermore, we found a positive relationship between right hippocampal volume at time 2 and concurrent BMIz. These results suggest that measured brain region volumes are associated with concurrent BMIz and BMIz change over one-year within a primarily healthy weight sample of children. Therefore, brain morphometry may be a useful predictive marker of longitudinal weight gain, particularly in children. Furthermore, as the entorhinal cortex integrates signaling from both reward and control regions of the brain our findings suggest that this region may be particularly important to regulating weight during child development. Future large-scale longitudinal neuroimaging studies are needed to further investigate the relationship between brain structures and BMIz in children.

## Supporting information

S1 Table Regressions from the SEM’s between BMIz scores and regional brain volumes at baseline and 1-year follow up in females. (DOCX)

S2 Table Regressions from the SEM’s between BMIz scores and regional brain volumes at baseline and 1-year follow up in males. (DOCX)
