# Supplementary material for: Association between regional brain volumes and BMI z-score change over one year in children
Source: PLoS One. 2019 Sep 19;14(9):e0221995. doi: 10.1371/journal.pone.0221995 (PMC6752809; doi:10.1371/journal.pone.0221995)
Supplement: S1 Table — (DOCX) [file pone.0221995.s001.docx]

**S1 Table.** Regressions from the SEMs between BMIz scores and regional brain volumes at baseline and 1-year follow up for females

| **Outcome** | **Brain Region Predictor** | **Model Estimate**  β | |
| --- | --- | --- | --- |
| BMIz at Time 1 |  | Left | Right |
|  | Nucleus Accumbens T1 | 0.02 | 0.35 |
|  | Entorhinal Cortex T1 | -0.10 | -0.27 |
|  | Hippocampus T1 | 0.05 | -0.009 |
| BMIz at Time 2 |  |  |  |
|  | Nucleus Accumbens T1 | -0.09 | -0.22 |
|  | Entorhinal Cortex T1 | **-0.19*** | **-028*** |
|  | Hippocampus T1 | 0.27 | **-0.20*** |
|  |  |  |  |
|  | Nucleus Accumbens T2 | -0.05 | -0.18 |
|  | Entorhinal Cortex T2 | **0.16*** | 0.05 |
|  | Hippocampus T2 | 0.03 | **0.44*** |

*Indicates p<0.05.

N=27 (males)

Models include control variables for estimated intracranial volume, age, sex, and pubertal status where significant

Volumes are reported as sample standardized z-scores = Volume – Sample Mean Volume / Sample Standard Deviation.

T1= Baseline; T2= 1 Year Follow Up.
