# Supplementary material for: Association between regional brain volumes and BMI z-score change over one year in children
Source: PLoS One. 2019 Sep 19;14(9):e0221995. doi: 10.1371/journal.pone.0221995 (PMC6752809; doi:10.1371/journal.pone.0221995)
Supplement: S2 Table — (DOCX) [file pone.0221995.s002.docx]

**S2 Table.** Regressions from the SEMs between BMIz scores and regional brain volumes at baseline and 1-year follow up for males

| **Outcome** | **Brain Region Predictor** | **Model Estimate**  β | |
| --- | --- | --- | --- |
| BMIz at Time 1 |  | Left | Right |
|  | Nucleus Accumbens T1 | -0.34 | -0.20 |
|  | Entorhinal Cortex T1 | -0.14 | 0.13 |
|  | Hippocampus T1 | -0.20 | 0.35 |
| BMIz at Time 2 |  |  |  |
|  | Nucleus Accumbens T1 | **-0.25*** | -0.02 |
|  | Entorhinal Cortex T1 | -0.04 | -0.08 |
|  | Hippocampus T1 | -0.004 | -0.20 |
|  |  |  |  |
|  | Nucleus Accumbens T2 | -0.04 | **-0.23*** |
|  | Entorhinal Cortex T2 | 0.08 | 0.12 |
|  | Hippocampus T2 | 0.15 | **0.41*** |

*Indicates p<0.05.

N=20 (females)

Models include control variables for estimated intracranial volume, age, sex, and pubertal status where significant

Volumes are reported as sample standardized z-scores = Volume – Sample Mean Volume / Sample Standard Deviation.

T1= Baseline; T2= 1 Year Follow Up.
